# Remission of Inflammatory Bowel Disease in Glucose-6-Phosphatase 3 Deficiency by Allogeneic Haematopoietic Stem Cell Transplantation

**DOI:** 10.1093/ecco-jcc/jjz112

**Published:** 2019-06-03

**Authors:** Chrissy Bolton, Nicola Burch, James Morgan, Beth Harrison, Sumeet Pandey, Alistair T Pagnamenta, Oxford IBD cohort investigators, Carolina Arancibia, Adam Bailey, Ellie Barnes, Beth Bird-Lieberman, Oliver Brain, Barbara Braden, Jane Collier, James East, Alessandra Geremia, Lucy Howarth, Simon Leedham, Rebecca Palmer, Astor Rodrigues, Alison Simmons, Peter Sullivan, Jenny C Taylor, John M Taylor, Judith C W Marsh, Victoria Potter, Simon Travis, Holm H Uhlig

**Affiliations:** 1 Translational Gastroenterology Unit, NIHR Oxford Biomedical Research Centre, Nuffield Department of Experimental Medicine, University of Oxford, John Radcliffe Hospital, Oxford, UK; 2 University Hospitals Coventry and Warwickshire NHS Trust, Coventry, UK; 3 Wellcome Centre for Human Genetics, University of Oxford, Oxford, UK; 4 Oxford NIHR Biomedical Research Centre, Oxford, UK; 5 Oxford Medical Genetics Laboratories, Oxford University Hospitals NHS Foundation Trust, Oxford, UK; 6 Department of Haematological Medicine, King’s College Hospital/King’s College London, London, UK; 7 Department of Paediatrics, University of Oxford, John Radcliffe Hospital, Oxford, UK

**Keywords:** Exome sequencing, inflammatory bowel disease, genomics, immunodeficiency

## Abstract

Mendelian disorders in glucose-6-phosphate metabolism can present with inflammatory bowel disease [IBD]. Using whole genome sequencing we identified a homozygous variant in the glucose-6-phosphatase *G6PC3* gene [c.911dupC; p.Q305fs*82] in an adult patient with congenital neutropenia, lymphopenia and childhood-onset, therapy-refractory Crohn’s disease. Because *G6PC3* is expressed in several haematopoietic and non-haematopoietic cells it was unclear whether allogeneic stem cell transplantation [HSCT] would benefit this patient with intestinal inflammation. We show that HSCT resolves *G6PC3-*associated immunodeficiency and the Crohn’s disease phenotype. It illustrates how even in adulthood, next-generation sequencing can have a significant impact on clinical practice and healthcare utilization in patients with immunodeficiency and monogenic IBD.

## 1. Introduction

Inflammatory bowel disease [IBD] is a chronic inflammatory condition triggered and perpetuated by a breakdown in mucosal homeostasis.^1^ For most patients with IBD, many genetic variants contribute a small risk of developing the disease, in line with a complex polygenic disorder.^2^ However in some patients, a single gene has a dominant impact on the risk of IBD.^1,3^ These ‘monogenic’ forms of IBD are often severe and therapy-refractory, with uncommon extra-intestinal manifestations and immunodeficiency.^4,5^ One immunodeficiency group is characterized by congenital neutropenia with persistent neutrophil counts under 0.5 × 10^9^/L, leaving patients vulnerable to recurrent or fatal infection.^6,7^ Among the 24 identified genes causing congenital neutropenia,^7^*WAS*, *SLC37A4* and *G6PC3* have been particularly associated with monogenic IBD.^5^

Therapeutic options for patients with congenital neutropenia include granulocyte-colony stimulating factor [G-CSF] to increase neutrophil counts and antimicrobials to manage infection.^8^ Whilst these treatments are standard of care to prevent neutropenia and infection, they often do not resolve intestinal inflammation.^9–14^

Allogeneic haematopoietic stem cell transplant [HSCT] is increasingly being used to treat patients with congenital neutropenia and other immune-mediated disorders of monogenic IBD.^15^

However, each condition needs to be evaluated, as the intestinal response to allogeneic HSCT is very variable amongst different causes of monogenic IBD. HSCT cures interleukin-10 (IL10) signalling defects causing IBD.^4^ In contrast, it does not improve intestinal inflammation in TTC7A deficiency,^16^ a disease manifesting with IBD, intestinal atresia and immunodeficiency. Similarly, in NEMO-deficient patients caused by defects in *IKBKG*, HSCT resolves the immunodeficiency but does not cure intestinal inflammation.^17^ The indication, concept and prognosis of allogeneic HSCT differs from the autologous HSCT trialled in classical IBD.^18,19^

As *G6PC3* is expressed in the cells of multiple organs and tissues, including non-haematopoietic cells such as epithelial cells and fibroblasts,^20^ the outcome of HSCT on intestinal inflammation requires confirmation. We present a patient with congenital neutropenia resulting from bi-allelic variants in the catalytic subunit-3 of glucose-6-phosphatase [*G6PC3*] whose markers of systemic inflammation and symptoms of therapy-resistant Crohn’s disease resolved with HSCT.

## 2. Case Presentation

From 14 months of age the male patient developed recurrent infections. He was diagnosed with congenital neutropenia and treated with G-CSF.

**Figure 1.**
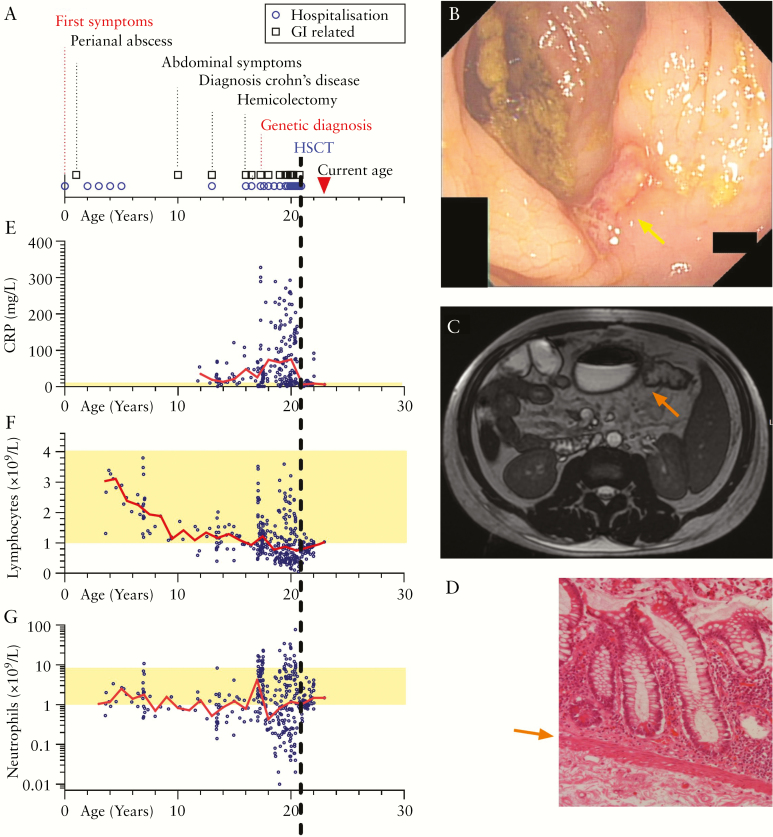
Phenotypic characteristics of a G6PC3-deficient patient with IBD. [A] Clinical course of the patient [symptoms, hospitalization, operations]. [B] Colonoscopy imaging of an impassable stricture at the anastomosis of the neo-terminal ileum illustrating deep ulceration [yellow arrow]. [C] Correlated cross-sectional axial magnetic resonance imaging scan showing colonic dilatation proximal to the stricture encountered in B [orange arrow]. [D] Haematoxylin and eosin staining of colonic biopsy showing inflammatory infiltrate. The muscularis mucosae is indicated [orange arrow]. [E–G] Biochemical results over time of CRP [mg/L, E], total lymphocyte count [F] and neutrophil counts [G] per 10^9^/L. The normal range [yellow band] and yearly median result [red line] are indicated. The timing of HSCT is illustrated by the dashed black line.

At 10 years of age the patient began losing weight, dropping to the 3^rd^ centile on growth charts. By age 13 years, he had developed abdominal pain, diarrhoea and mouth ulcers [Figure 1A]. On colonoscopy he was found to have patchy colonic and terminal ileal inflammation with stricture formation, leading to a diagnosis of Crohn’s disease. Given his immunodeficiency, the intestinal inflammation was managed conservatively with an elemental diet, nasogastric feeding and steroids, which was initially successful.

At 16 years of age, the patient developed intestinal obstruction secondary to a fibrotic stricture and inflammatory mass in his transverse colon [Figure 1B, C]. He required parenteral nutrition and underwent strictureplasties and an extended right-hemicolectomy with ileocaecal resection at 17 years of age. Histological reports confirmed inflammation with lymphocytic infiltration consistent with Crohn’s disease [Figure 1D].

One year post-operatively pain and diarrhoeal symptoms returned, with recurrent neutropenic sepsis. Gastrointestinal inflammation did not resolve with adalimumab (anti-tumour necrosis factor [anti-TNF]) or vedolizumab [alpha-4-beta-7 integrin antagonist] despite dose escalation, adequate therapeutic levels and a lack of anti-biologic antibody detection. Colonoscopy showed a fibrotic stricture at the ileo-colonic anastomosis. The patient was steroid-, antibiotic- and G-CSF-dependent. Unfortunately, his condition deteriorated, resulting in 25 admissions and >130 blood tests over 2 years for neutropenic sepsis and abdominal pain [Figure 1E–G]. Eventually he was being admitted every 2–3 weeks, with C-reactive protein [CRP] raised over a long period. As a neutrophil-derived protein, faecal calprotectin was not reliable as a marker of intestinal inflammation in this patient with repeat episodes of neutropenia. Peak faecal calprotectin values of 276 mg/kg stool corresponded to intestinal inflammation during times of normal or increased neutrophil counts, whereas normal levels of faecal calprotectin were noted during times of neutropenia [false negative test]. Immunophenotyping revealed a progressive lymphopenia, in particular T cell lymphopenia [0.49 × 10^9^/L] with low levels of CD8 cells [0.15 × 10^9^/L], NK cells [0.02 × 10^9^/L] and high IgG [16.6 g/L].

**Figure 2.**
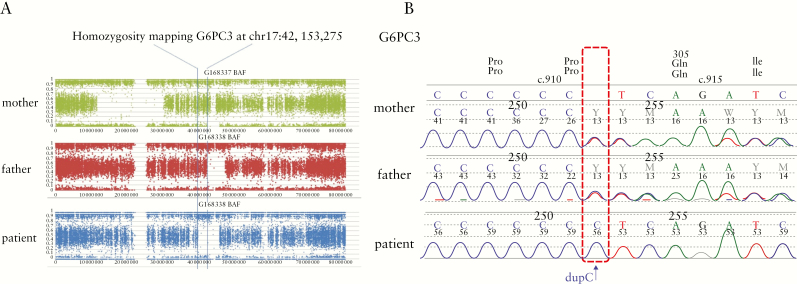
Genetic analysis. [A] Allelic ratios shown for the patient and his parents across chromosome 17. The fraction of reads supporting the non-reference base vs the total number of reads at that locus is plotted for 106 316 variants along chromosome 17. This set of variants was chosen because they were high confidence [i.e. annotated with PASS flag by variant caller, single-nucleotide variant not indels, only one non-reference allele and present in single nucleotide polymorphism database] and had coverage between 15 and 100× in all three subjects. The *G6PC3* variant lies in a >5-Mb region of autozygosity in the proband, the distal portion of which is also shared autozygously in the unaffected father. [B] Sanger sequencing of the patient with homozygous *G6PC3* c.911dupC in comparison to his parents.

At 18 years of age, he consented to genomic sequencing. This analysis revealed a rare homozygous c.911dupC; p.Q305fs*82 alteration in *G6PC3* [NM_138387.3] within a >5-Mb region of homozygosity [Figure 2]. The parents of this patient are both heterozygous carriers of c.911dupC, without known consanguinity. The same c.911dupC indel/frameshift variant has been described previously in a patient with congenital neutropenia and an atrial septal defect.^21^

With severe pain, neutropenic sepsis and frequent hospitalization, conventional treatment strategies were exhausted and allogeneic HSCT was undertaken. Aged 20 years, in the absence of a matched sibling or matched unrelated donor, he underwent haploidentical HSCT from his father. A reduced intensity conditioning regimen of fludarabine 30 mg/m^2^ daily was used from day −6 to −2, cyclophosphamide [CY] 14.5 mg/kg on days –6 and –5, and total body irradiation of 2 Gy on day –1. Post-graft immunosuppression involved high-dose CY 50 mg/kg on days +3 and +4, mycophenolate mofetil [day +5 to +35] and tacrolimus from day +7 continuing for 12 months.^22^ Neutrophil and platelet engraftment occurred on days +15 and +17, respectively. At 3 months there was full donor myeloid chimerism [CD15 99%] and mild mixed T-cell chimerism [CD3 92%] with subsequent full donor T-cell chimerism. His post-transplant course was uneventful apart from grade II graft-versus-host disease of the skin that did not require systemic treatment.

Almost immediately the patient’s pain and gastrointestinal symptoms dramatically improved. He weaned off opiate analgesia, his neutropenia and lymphopenia resolved and he has not been hospitalized again in 2.5 years of follow-up. His body mass index increased from a range of 12.5–17.10 pre-transplant to 19.9 and 23.6 in the first and second year, respectively, post-transplant. His CRP reduced from a yearly median of 76 mg/L [interquartile range 17–148 mg/L] pre-transplant to 7 mg/L [6–24 mg/L] post-transplant [Figure 1E]. Intestinal obstructive symptoms resolved and artificial nutrition could be stopped, with no further treatment needed for IBD. Because his clinical symptoms had so markedly improved, the patient did not consent to any further imaging or colonoscopies.

## 3. Discussion

We describe the first case of HSCT-mediated clinical remission of Crohn’s disease in a patient with a pathogenic *G6PC3* gene variant. The patient exhibited congenital neutropenia, lymphopenia, NK cell loss and IBD, which responded to allogeneic HSCT at the age of 20 years.

G6PC3 is required for glucose homeostasis of cells, where it catalyses the hydrolysis of glucose-6-phosphate [G6P] to glucose within the endoplasmic reticulum [ER].^23^ Although *G6PC3* is expressed ubiquitously,^20^ neutropenia is a consistent finding in patients with G6PC3 deficiency.^24^ The related syndrome glycogen storage disorder-1b [GSD-1b] also manifests neutropenia and enterocolitis^24^ and is caused by defects affecting the G6PC3-coupled glucose-6-phosphate transporter. In both disorders, defective G6P metabolism causes an increased propensity towards cellular apoptosis.^25^ Granulocytes rely on anaerobic glycolysis for energy generation and are unable to utilize compensatory gluconeogenesis.^24,26^ With limited activity of glucose-dependent pathways, NADPH, lactate and ATP substrates are reduced.^23^ The effects on energy generation, increased ER stress and impaired superoxide production may contribute to myeloid cell dysfunction.^14,23,27^

IBD-like colitis has been described in at least 8% of patients with G6PC3 deficiency.^14,24^ Patients present with Crohn’s-like inflammation, frequent stricture formation and severe oral aphthous ulceration.^12–14,24^ Myeloid cells are particularly dependent on G6PC3, lacking compensatory alternative phosphatases [Supplementary Figures 1–3]. Hence, colitis in G6PC3 deficiency is attributed to haematopoietic cell defects, rather than intestinal epithelial or other non-haematopoietic cell defects. Defective antimicrobial activity by the innate immune system is one mechanism proposed to underlie colitis in G6PC3-deficient patients. This impairment may arise from the altered survival and function of myeloid-lineage cells seen in G6PC3 deficiency.^24,27^ Neutrophils deficient in G6PC3 show increased rates of apoptosis and early arrest of maturation.^23,24,27^ G6PC3-deficient myeloid cells demonstrate diminished respiratory burst, impaired superoxide production, chemotaxis and phagocytosis,^23,24^ which may also contribute to the defective antimicrobial immune response.

Autoinflammation occurring through activation of the inflammasome has also been proposed as an driver of IBD,^14^ with G6PC3-deficient neutrophils producing significantly increased levels of pro-inflammatory cytokines in response to lipopolysaccharide.^14^ As rates of apoptosis in G6PC3-deficient neutrophils are consistently enhanced upon TNF-α stimulation,^27^ pro-inflammatory states may perpetuate neutropenia. In addition, our case and a number of colitic G6PC3-deficient patients exhibited lymphopenia with low levels of recent thymic-emigrant T lymphocytes [CD4+CD31+CD45RA+ T cells].^12^ Our patient exhibited 12.1% of CD+CD31+CD45RA+ T cells [normal range 19.2–60%].

G-CSF is often effective in normalizing neutrophil counts,^27^ although in some patients, G-CSF therapy may be unable to control intestinal inflammation or correct neutropenia.^13,15,28^ In keeping with the metabolic component of the disease in patients with GSD-1b disease, liver transplant has been trialled to improve metabolic homeostasis and hypoglycaemia.^29^ Unexpectedly, neutropenia improved in 64% of transplanted patients in one study,^29^ but IBD-related outcomes and the genetic status of the patients were not reported.

Two patients with G6PC3 deficiency have undergone HSCT for refractory neutropenia.^12,15^ The presence or outcome of IBD in these patients is not known [F. Fioredda, pers. comm.]. Whilst the curative potential of HSCT for congenital neutropenia patients is an exciting prospect, it is not undertaken lightly given the potential for adverse effects or mortality. In HSCT of 136 patients with congenital neutropenia, the cumulative incidence of acute graft-versus-host disease was 21%, with 17% transplant-related mortality.^15^

HSCT has been associated with better outcomes in congenital neutropenia when administered at a younger age,^15^ but the present case illustrates the clinical benefit of a genetic diagnosis and the life-transforming potential of allogeneic transplantation in adulthood. Further studies are required to evaluate the efficacy and safety of HSCT as a treatment for neutropenia and IBD in G6PC3 deficiency. This patient illustrates the importance of whole genome sequencing, where curative precision medicine for immununodeficiency and IBD may be offered on the basis of the molecular diagnosis.

## 4. Materials and Methods

### 4.1. Patient

The patient was recruited to the prospective Oxford IBD Cohort. The study was approved by the local ethics committee [Inflammatory Bowel Disease in Oxford: prospective cohort for outcomes, treatment and predictors. Research Ethics Committee Reference: 09/H1204/30.

### 4.2. Genome sequencing

For whole genome sequencing, parent–child trio samples were prepared using an Illumina TruSeq DNA PCR-free library preparation kit and sequenced using an Illumina HiSeq 2500 device [read length 2 × 100]. Reads were mapped to hg19 and variants were called with Isaac v.2.0.17 [Illumina].

Sanger sequencing for genotype validation was performed using standard techniques.

### 4.3. Variant screening

To prioritize rare IBD-associated variants of clinical significance, 59 genes associated with Mendelian forms of IBD were screened.^5,30^ We focused on rare deleterious or potentially deleterious variants that had minor allele frequency < 1% and were consistent with the reported inheritance pattern of the given gene [as previously summarized^30^].

We screened VCF files manually using custom scripts and with VariantStudio v2.2 [Illumina]. We investigated functional variants with transcript ablation, stop gained/lost, stop retained, splice donor/acceptor/region, frameshift, inframe insertion/deletion, initiator codon and missense variants with a PolyPhen-2 and SIFT pathogenicity predictions of ‘possibly damaging/deleterious’ [or greater]. A secondary analysis was performed using Ingenuity Variant Analysis [Qiagen].

## Conflict of Interest

None of the authors have a conflict of interest related to this article. HHU received research support or consultancy fees from Eli Lilly, UCB Pharma, Celgene, Boehringer Ingelheim, Pfizer and AbbVie. SPLT has been adviser to, in receipt of educational or research grants from, or invited lecturer for AbbVie, Amgen, Asahi, Biogen, Boehringer Ingelheim, BMS, Cosmo, Elan, Enterome, Ferring, FPRT Bio, Genentech/Roche, Genzyme, Glenmark, GW Pharmaceuticals, Immunocore, Immunometabolism, Janssen, Johnson & Johnson, Lilly, Merck, Novartis, Novo Nordisk, Ocera, Pfizer, Shire, Santarus, SigmoidPharma, Synthon, Takeda, Tillotts, Topivert, Trino Therapeutics with Wellcome Trust, UCB Pharma, Vertex, VHsquared, Vifor, Warner Chilcott and Zeria. JM has received research funding from Novartis and honoraria from Novartis, Alexion and Jazz.

## Supplementary Material

jjz112_suppl_Supplementary_File
