# Supplementary material for: Remission of Inflammatory Bowel Disease in Glucose-6-Phosphatase 3 Deficiency by Allogeneic Haematopoietic Stem Cell Transplantation
Source: J Crohns Colitis. 2019 Jun 3;14(1):142–7. doi: 10.1093/ecco-jcc/jjz112 (PMC6930000; doi:10.1093/ecco-jcc/jjz112)
Supplement: jjz112_suppl_Supplementary_File [file jjz112_suppl_supplementary_file.docx]

**Remission of inflammatory bowel disease in Glucose-6-Phosphatase 3 deficiency by allogeneic haematopoietic stem cell transplantation**

Chrissy Bolton^1^*, Nicola Burch^2*^, James Morgan^2^, Beth Harrison^2^, Sumeet Pandey^1^, Alistair T. Pagnamenta^3, 4^, Oxford IBD cohort investigators^¶^, Jenny C. Taylor^3, 4^, John M. Taylor^4, 5^, Judith C. W. Marsh^6^, Victoria Potter^6^, Simon Travis^1, 4^, Holm H. Uhlig^1, 4,7**^

**Supplementary File**

**Supplementary Figure 1:**

**Differential expression of different Glucose-6-Phosphatases (GCPC3, G6PC2 and G6PC).**


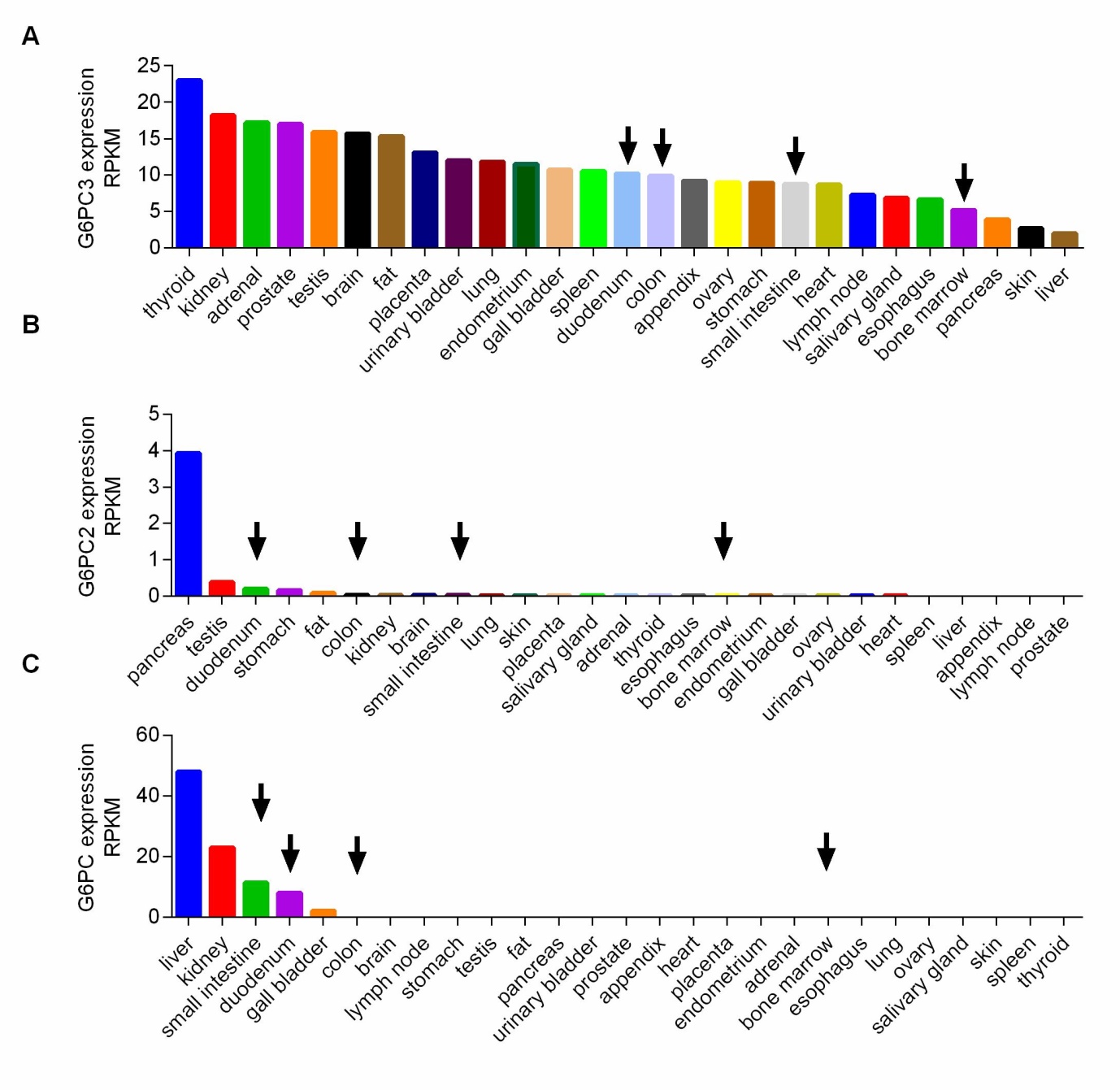


(A-C) mRNA expression data for different Glucose-6-Phosphatases (GCPC3 [A], G6PC2 [B] and G6PC [C] was extracted from available RNA-seq data of tissue samples from 95 human individuals representing 27 different tissues (BioProject: PRJEB4337 <https://www.ncbi.nlm.nih.gov/gene/>^31,32^). Black arrows in each graph indicate intestinal tissue and bone marrow. Data is shown as Reads Per Kilobase of transcript per Million mapped reads (RPKM)^31^.

**Supplementary Figure 2: Differential expression of different Glucose-6-Phosphatase (GCPC3, G6PC2 and G6PC) in the sorted primary epithelial cells.**


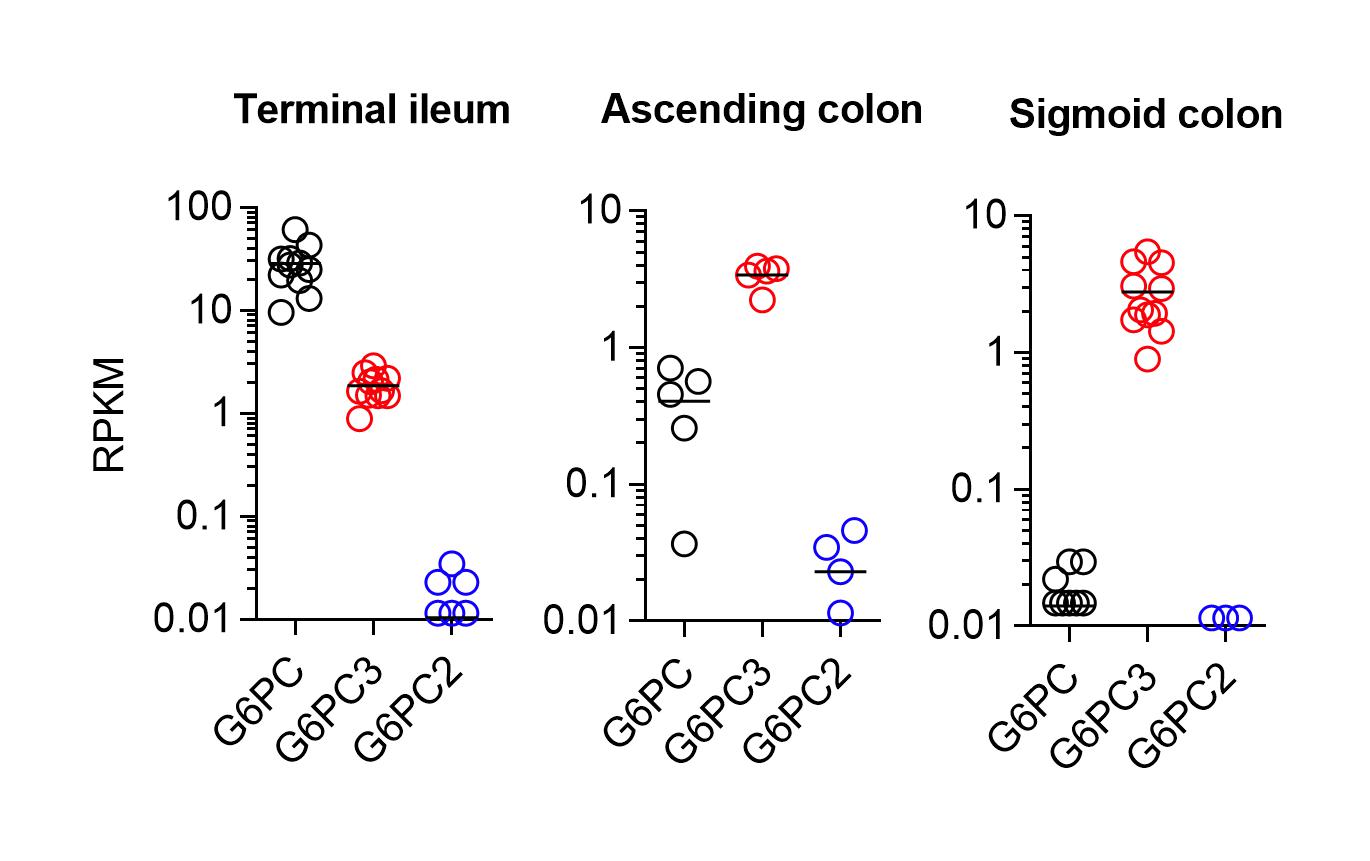


mRNA expression data for different Glucose-6-Phosphatases (GCPC3, G6PC2 and G6PC) were extracted from publically available RNA-seq data of paediatric biopsies samples (Expression Atlas - E-MTAB-5464 <https://www.ebi.ac.uk/gxa/home>^33,34^). Data is shown as Reads Per Kilobase of transcript per Million mapped reads (RPKM)^34^.

**Supplementary Figure 3**: **G6PC3 expression across different primary immune cells**





Gene expression data of G6PC3 across different primary immune cell population was extracted from the BLUEPRINT Epigenome project (<https://www.ebi.ac.uk/gxa/home>^33,35^). Data is shown as Transcript Per Million (TMP)^35^.

**Supplementary References**

31. Fagerberg L., Hallström BM., Oksvold P., Kampf C., Djureinovic D., Odeberg J., et al. Analysis of the Human Tissue-specific Expression by Genome-wide Integration of Transcriptomics and Antibody-based Proteomics. *Mol Cell Proteomics* 2014;**13**(2):397–406. Doi: 10.1074/mcp.M113.035600.

32. National Centre for Biotechnology Information. Available at: https://www.ncbi.nlm.nih.gov/bioproject/231263. Accessed May 19, 2019.

33. The European Bioinformatics Institute. EMBL-EBI. Available at: https://www.ebi.ac.uk/. Accessed April 17, 2019.

34. Howell KJ., Kraiczy J., Nayak KM., Gasparetto M., Ross A., Lee C., et al. DNA Methylation and Transcription Patterns in Intestinal Epithelial Cells From Pediatric Patients With Inflammatory Bowel Diseases Differentiate Disease Subtypes and Associate With Outcome. *Gastroenterology* 2018;**154**(3):585–98. Doi: 10.1053/j.gastro.2017.10.007.

35. Adams D., Altucci L., Antonarakis SE., Ballesteros J., Beck S., Bird A., et al. BLUEPRINT to decode the epigenetic signature written in blood. *Nat Biotechnol* 2012;**30**(3):224–6. Doi: 10.1038/nbt.2153.
